# Plasmonic Bowl-Shaped Nanopore for Raman Detection of Single DNA Molecules in Flow-Through

**DOI:** 10.1021/acs.nanolett.3c00340

**Published:** 2023-06-01

**Authors:** Yingqi Zhao, Aliaksandr Hubarevich, Angela Federica De Fazio, Marzia Iarossi, Jian-An Huang, Francesco De Angelis

**Affiliations:** † Istituto Italiano di Tecnologia, Via Morego 30, 16163 Genova, Italy; ‡ Faculty of Medicine, Faculty of Biochemistry and Molecular Medicine, University of Oulu, Aapistie 5 A, 90220 Oulu, Finland

**Keywords:** plasmonic nanopore, surface-enhanced Raman spectroscopy, DNA detection, flow-through, nanofluidic molecular trapping

## Abstract

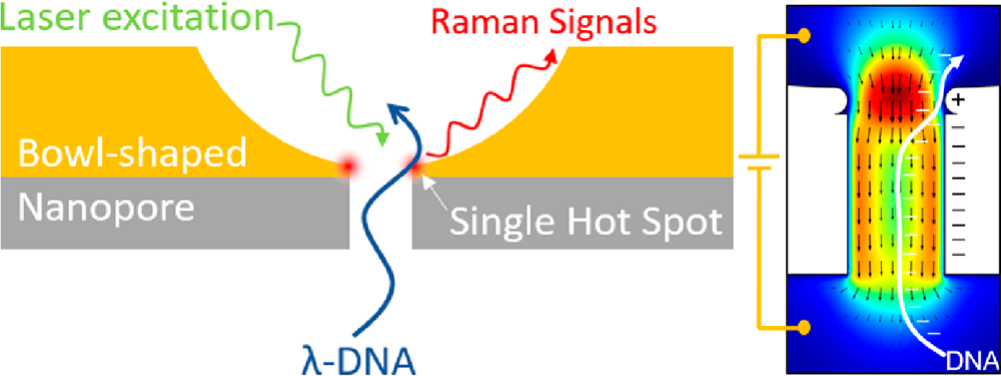

Plasmonic nanopores combined with Raman spectroscopy are emerging as platforms for single-molecule detection and sequencing in label-free mode. Recently, the ability of identifying single DNA bases or amino acids has been demonstrated for molecules adsorbed on plasmonic particles and then delivered into the plasmonic pores. Here, we report on bowl-shaped plasmonic gold nanopores capable of direct Raman detection of single λ-DNA molecules in a flow-through scheme. The bowl shape enables the incident laser to be focused into the nanopore to generate a single intense hot spot with no cut off in pore size. Therefore, we achieved ultrasmall focusing of NIR light in a spot of 3 nm. This enabled us to detect 7 consecutive bases along the DNA chain in flow-through conditions. Furthermore, we found a novel electrofluidic mechanism to manipulate the molecular trajectory within the pore volume so that the molecule is pushed toward the hot spot, thus improving the detection efficiency.

Nanopore sequencing capable of resistive pulse long-read of DNA molecules in a portable device is playing an important role in personalized medicine and digital health.^1−5^ To extend it to protein detection and sequencing, various types of solid-state nanopores are being developed to include optical and electrical detection methods as well as stability in different physiological environments.^2,5−11^ In particular, plasmonic nanopores are emerging as a promising platform for both protein and nucleic acid detection and, in the longer term, sequencing.^10,12−15^

Recently, we have developed a plasmonic particle-in-pore sensor^16^ that adsorbed peptides onto a gold nanoparticle and then successively flowed and trapped them into the plasmonic pore. The gap formed between the particle surface and the pore wall offered a strong plasmonic field, while the trapping increased the detection time. Under these favorable conditions, single amino acids were detected and discriminated in single peptides by surface-enhanced Raman spectroscopy (SERS).^17^ Notably, all 20 proteinogenic amino acids were discriminated even at the level of single residues in single peptides.^18^ However, the particle-in-pore approach can hardly be translated to practical applications. In this regard, it would be ideal to directly translocate the analyte into a plasmonic nanopore without absorbing it onto a particle. To reach this target, different obstacles must be cleared.

The first point regards the size of the pore which should have a diameter as small as possible to force the molecule to pass through the pore “longitudinally”. As is well-known, in pores with a diameter greater than 5 nm, 60–70% of the molecules can translocate with folded conformation, which can be detrimental to the identification process.^19^ Also, plasmon intensity decays very fast away from the metallic surface. Therefore, the plasmonic field does not fill the whole pore volume unless the diameter approaches the decay length that, in the visible range, is a few nm. Hence, the molecule can translocate without being detected, for instance, by passing in the center of the pore where the plasmonic field is low or even vanishing. In this regard, ideal plasmonic pores should have a diameter of 3–4 nm, which is quite challenging to fabricate. Furthermore, such a small pore does not necessarily show strong field enhancement. In fact, the small pore radius would lead to lower plasmonic enhancement, thus limiting Raman sensitivity, as we will better explain later.

Second, to correctly identify submolecular features and their position within the molecule is essential because the probing field is unique. For example, cylindrical pores or porous materials provide multiple hot spots along the molecule trajectory that will generate multiple Raman emissions. As a result, the corresponding data set can be so complex to analyze that it can lead to misinterpretation of the molecular structure, sequence, or identity. One should consider that Raman signals at the single-molecule level can be very weak and noisy. Any factor that makes them even more complex, noisy, or weaker may represent a significant obstacle. Therefore, the ideal plasmonic pore should provide a single hot spot with a size as small as comparable to the size of a single or a few amino acids and the intensity as high as possible.

In this paper, we show that 3D bowl-shaped plasmonic nanopores combined with a new trapping mechanism present a significant step forward to clear the mentioned obstacles. First, the bowl-shaped structure focuses the optical energy into the nanopore orifice to generate a single hot spot with strong enhancement and no cutoff in pore size. Hence, in opposition with conventional nanopores, the smaller the pore, the higher the enhancement. Even though the current fabrication limits do not allow the production of ultrasmall metallic pores, we show that a pore of 20 nm in diameter provides a unique plasmonic hot spot of 3 nm in linear size. Notably, the combination of hot spot intensity and reduced spatial size enables us to detect small portions of DNA molecules down to 7 consecutive nucleotides, a performance not far from that achievable with electrical readouts.

Furthermore, long-lasting DNA trappings up to tens of seconds were observed repeatedly. By taking advantage of numerical computations, we explained such a long trapping time through a balance between electro-osmotic sheath flow and electrophoretic forces. The balance occurs under specific constraints for the pore shape, size, and materials, and it resembles effects happening in bipolar electrodes characterized by a steep variation of the surface charge at the interface between two materials.^20,21^ The results of all these effects enable us to record strong Raman signals from DNA molecules flowing into nanopores without the use of a particle-in-pore approach^17^ or extra labeling. However, the approach is suitable for both nucleic acids and proteins, even though for the latter specific delivering protocols must be developed.^22^

The device is shown in Figure 1A. We fabricated bowl-shaped nanopores on a silicon nitride membrane (Figure 1 A, B, C) by a focused ion beam (FIB) system. The bowl shape was achieved by sculpturing the gold film of 100 nm in thickness with a group of concentric ring patterns starting from 150 nm diameter. The milling depths of each ring were tuned to form a bowl-shaped profile. Then, an additional gold layer of 10 nm thickness was sputtered on the front side of the nitride to further reduce the nanopore diameter. The λ-DNA moves as a random coil in solution with a gyration radius of 600 nm.^23^ Hence, to uncoil the molecules,^24^ agarose hydrogel with 200 nm pores on average was placed below the nanopore. The agarose hydrogel solution was dropped into the low chamber by a pipet and then cooled down according to our previous protocol.^25^ The nanopore was then encapsulated in a microfluidic chamber made from polydimethylsiloxane (PDMS). All the fabrication details, geometric parameters, and uncoiling principle are reported in the Supporting Information (Note 1).

**Figure 1:**
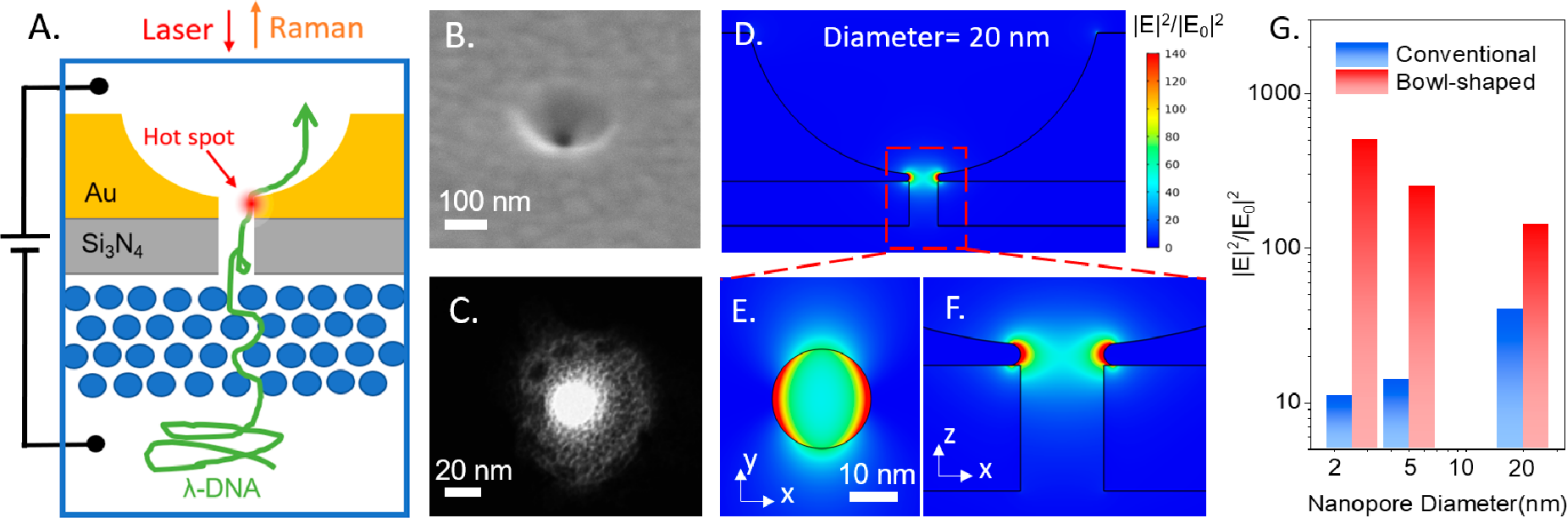
Schematic of the bowl-shaped plasmonic nanopore system. (A) Upon laser illumination with DC electric bias, the nanopore would generate a single plasmonic hot spot and trap an unfolded DNA segment (green arrow line) in the hot spot for SERS detection. (B) SEM image and (C) TEM image of the FIB-fabricated nanopore. (D) The simulated electric field intensity distribution of a bowl-shaped nanopore at wavelength 785 nm. (E) Magnified top view and (F) magnified side view. (G) Electric field intensity in the conventional cylindrical nanopore and bowl-shaped nanopore. The gold thickness at the pore is 5 nm.

The plasmonic response of the pores has been calculated by using COMSOL Multiphysics, and all the details are reported in the Supporting Information (Note 2) in which we give a complete comparison between bowl-shaped and conventional nanopores. According to the simulation results, the field enhancement of a 20 nm bowl-shaped nanopore approaches the remarkable value of 140 near the gold wall (Figure 1D, E, F). These values are enough to reach single-molecule sensitivity in SERS detection. Notably, as shown in Figure 1G when the radius of the pore is progressively reduced, the bowl-shaped pore shows a progressive enhancement of the field, while the conventional pores show a progressive reduction. A similar trend can be observed when the thickness of the gold layer is reduced below the skin depth (more details in Figures S3, S4, and S5).

This ability of focusing the electromagnetic radiation in a region with an extremely small volume resembles what happens in the adiabatic compression of surface plasmon polaritons in metallic tips or conical metallic waveguides.^26,27^ In this effect, plasmons excited at the base of metallic nanocones propagate toward the cone tip. During the propagation, the effective index of refraction increases, thus reducing the plasmon wavelength and enabling the plasmon wave to access the metallic tip even though the size of the tip is a few nm, i.e., hundreds of times smaller than the photon wavelength in the visible range. Such an ultra-sub-wavelength confinement has no cutoff regarding the tip size. The mechanism is known as adiabatic compression of plasmons.^28^ Its importance comes from the fact that, during propagation from the cone base to the tip, the optical energy is progressively squeezed in a smaller volume, thus increasing the local energy density. The latter results in a strong local amplification of the electromagnetic field at the tip apex. Since there is no cutoff with respect to the tip size, the smaller the tip the higher the field enhancement at the tip apex.

The bowl-shaped pores behave like an inverse conical waveguide able to deliver the energy into the pore that is the equivalent of the tip apex in a real conical waveguide. In other words, the pores show the same optical behavior, thus enabling huge field enhancements in an extremely reduced volume. As anticipated in the introduction, it is very important to generate hot spots with size and intensity capable of probing submolecular features such as single nucleotides or amino acids.

For example, when the pore radius is 2.5 nm and the metal thickness at the pore is 5 nm, the bowl-shaped nanopore provides a field intensity enhancement approaching 200 at λ = 785 nm in the pore center. Indeed, it is even much stronger along the pore walls. Notably, such a nanometric pore could satisfy all the requirements for detecting small molecules passing through. In fact, not only is the intensity very high but also the extension of the plasmonic field is approximately 3 nm along the direction of the translocation. Indeed, since the Raman intensity scales with the fourth power of the local field, the real probing region should be even smaller and hence comparable with a single nucleotide or amino acid. The state-of-the-art FIB systems such as Helium FIB Microscopes could produce nanometric gold pores. However, compatibly with our fabrication capability (FIB, Gallium Source, FEI Nova Nanolab 650, year 2011), we focused this work on pores of 20 nm in diameter. Even for this diameter, the bowl-shaped pore provides a unique probe field with a local intensity that is 5 times the one provided by conventional pores.

To test the ability of bowl-shaped pores to provide such an ultrasmall probing field, we conducted Raman measurements on the translocation of λ-DNAs through the bowl-shaped nanopore of different diameters under DC electric bias, as shown in Figure 1A. To prevent DNA from attaching on the nanopore surface, the pores were coated with a monolayer of 4-aminobenzenethiol (4-ABT) molecules. Then, λ-DNAs in 1 M LiCl electrolyte were dropped into the lower chamber and translocated through the nanopore under 30 mV bias across the nanopore upon 785 nm laser illumination. Examples of time traces of SERS spectra are reported in Figure 2 for the bowl-shaped pores of 20 nm.

**Figure 2:**
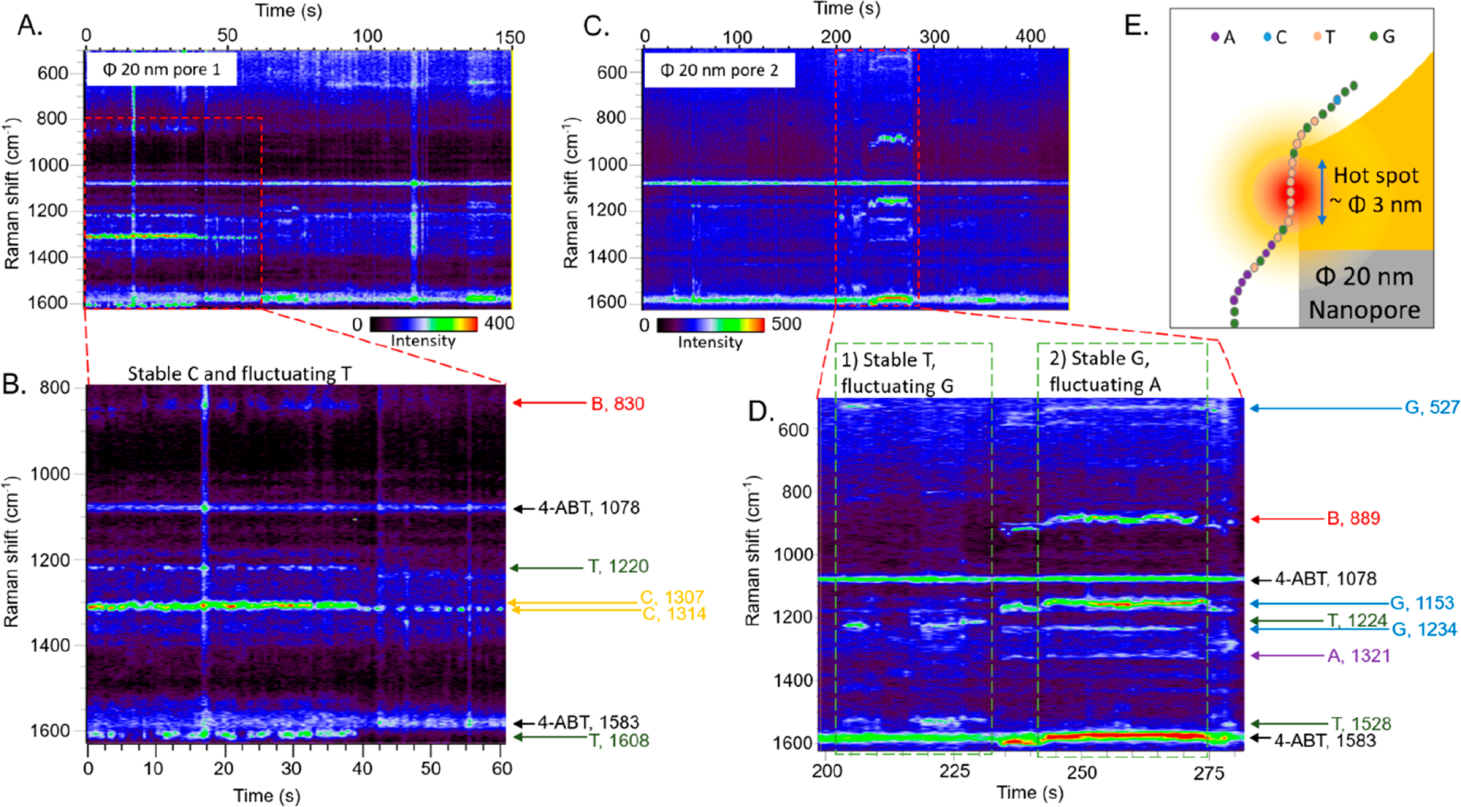
Time traces of SERS spectra of single-file λ-DNA that passes through the 20 nm diameter bowl-shaped nanopores with hydrogel under an electric bias of 30 mV in 1 M LiCl. The arrows are peak assignments where long arrows indicate stably trapped bases. (A) Full time trace of SERS spectra in pore 1. (B) The time trace of SERS spectra during 0–40 s in pore 1 showing a DNA segment with stable C and fluctuating T. (C) Full time trace of SERS spectra in pore 2. (D) The time trace of SERS spectra during 200–275 s in pore 2 showing DNA segments with (1) stable T and fluctuating G and (2) stable G and fluctuating A. (E) Schematic of the linearized λ-DNA and the single hot spot.

First, it is worth noticing that we observed strong and stable SERS signals of DNA bases,^29^ adenine (A), thymine (T), cytosine (C), guanine (G), as well as of the deoxyribose and phosphodiester backbone (B) of DNA.^30^ Their SERS band assignments are in the Supporting Information (Note 5). For example, C, T, and B bands were observed for 40 s in Figure 2B, and A, G, and B bands appear for 45 s in Figure 2D. Importantly, the background SERS signals of 4-ABT did not overlap with those of DNA bases. The quality of achieved signals and the signal-to-noise ratio demonstrated the ability of bowl-shaped pores to generate strong plasmonic hot spots.

Second, most of the times we observed continuous peaks of only 2 noncomplementary bases in which the signal from one base is stable for a long time while the one coming from the second base is fluctuating. For instance, in Figure 2B, continuous peaks of only 2 noncomplementary C and T bases were observed in the time region of 0–40 s. In this time interval, the C signal is constantly observed, while the T signal is fluctuating. Also, in another pore in Figure 2D, in the time region during 240–275 s, we observed stable peaks of the backbone and the 2 noncomplementary A and G bases. Similarly, the signal coming from G is stable, while the A peak is fluctuating. This particular behavior is compatible with trapping (in close proximity to the plasmonic hot spot) of short linear segments of the λ-DNA, such as TCCCCCT, GTTTTTTTG, or AGGGGGA, as shown in Supporting Information Table S5. For the sake of clarity, a possible configuration is sketched in Figure 2E in which the segment GTTTTTTTG is trapped near the pore wall. During the trapping period, the segment fluctuates around an average position due to the Brownian motion. As long as the amplitude of the Brownian fluctuations is lower than the segment size, one should expect that the signal coming from T bases is stable. In fact, the number of T bases probed by the plasmonic spot is weakly affected by the fluctuations of the segment around the average position. In opposition, the signal from G bases fluctuates because their spatial overlap with the plasmonic hot spot is not constant due to the segment fluctuations.

Notably, from these data we can estimate the spatial extension of the plasmonic hot spot that must be comparable to the length of the segments in the Supporting Information Table S5. Since one base has a length of 0.34 nm on average, the size of the hot spot ranges from 2.38 nm (7 bases) to 3.06 nm (9 bases) as shown in Figure 2E. This is in perfect agreement with the experimental value of the final thickness of the gold layer into the pore (approximately 5 nm) and the corresponding size of the hot spot calculated in the simulations (Figure 1 D, E, F).

Furthermore, the fact that we do not observe complementary bases further suggests that the pore is probing a single strand of DNA, although we employed double-stranded DNA. This is compatible with complete unfolding of DNA molecules as it is expected by the concomitant action of the agarose gel and the strong electrophoretic field in proximity to and inside the pore. Please note that the plasmonic hot spot extends all around half of the pore circumference as shown in Figure 1E. Hence, if the DNA translocates in a folded conformation, a different portion of the molecule should have been exposed to the plasmonic field. As a result, the corresponding signal should have contained a random distribution of bases, which is not what we have observed.

Questions may arise around the fact that the second strand of DNA is never observed. In fact, this is not surprising since the plasmonic field is known to rapidly vanish from the metallic surface. The plasmonic field acting on the strand that is closer to the metallic surface will be higher than the one acting on the strand that is further. Since Raman intensity is strongly nonlinear, the signal of the closer strand dominates on the signal of the other strand. This finding is in agreement with our simulations in which the plasmonic field significantly drops 2 nm from the metal surface. Half of this region is occupied by the layer of 4-ABT (0.6 nm) and the electric double layer (0.3 nm). The remaining region is occupied by one strand of DNA as observed in the Raman spectra.

To further check the behavior of the system with respect to the pore radius, we carried out Raman experiments with pores of larger size, namely, 45 nm in diameter. The results are reported in Figure 3. As expected, larger pores exhibit lower signal quality, and in opposition with the 20 nm pores, the complementary bases of the λ-DNA are observed. For example, signals of complementary G and C peaks appear in the period 0–100 s in Figure 3A. Hence, we can suppose that in large pores DNA molecules can translocate in a folded or partially folded conformation. Likely, even if the molecule unfolds during the translocation into the gel layer, it tends to refold immediately after coming out of the gel and entering the 45 nm pore. Also, the electrophoretic field is lower for larger pores, thus reducing its contribution to the unfolding process.

**Figure 3:**
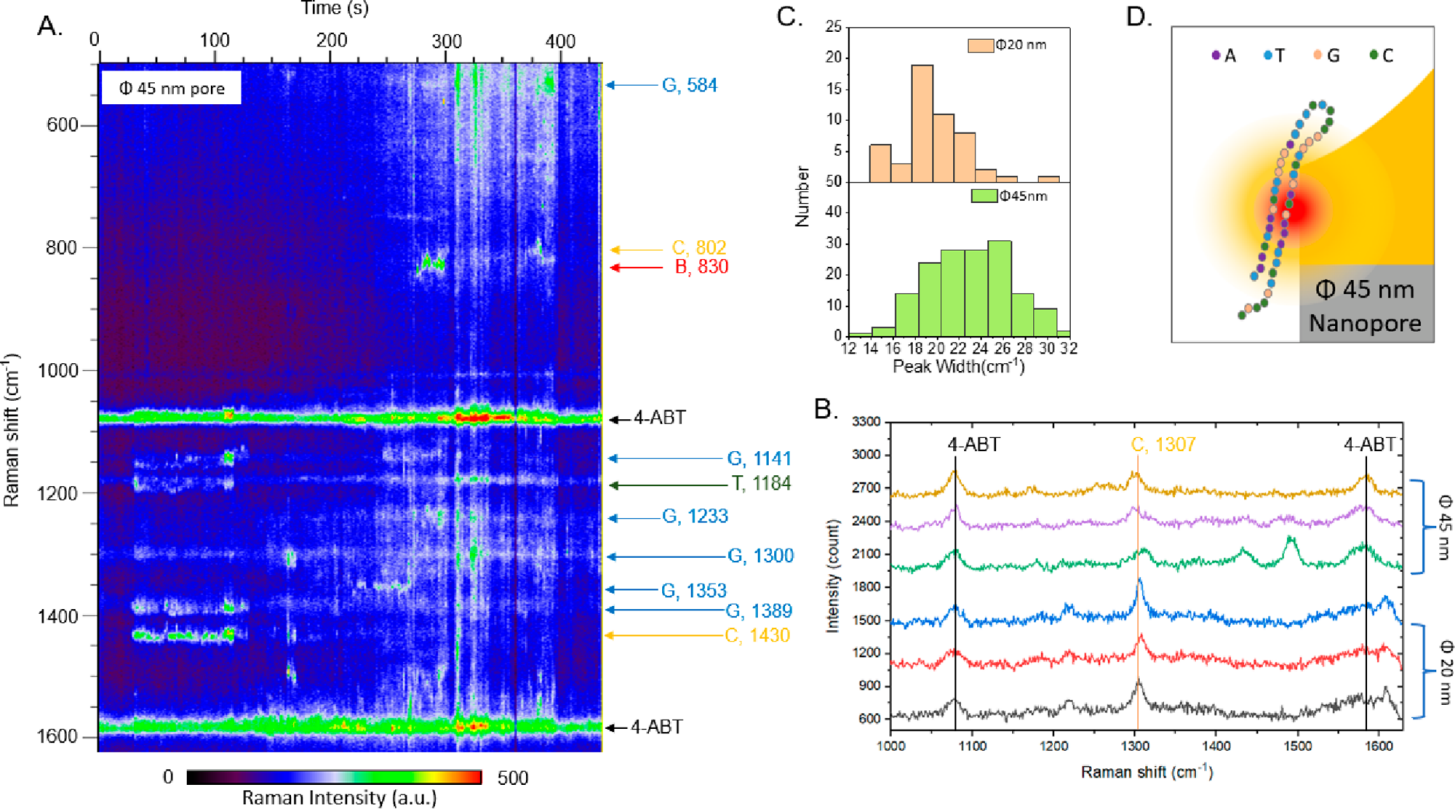
Time traces of SERS spectra of λ-DNA that pass through the 45 nm diameter bowl-shaped nanopore with hydrogel under an electric bias of 30 mV in 1 M LiCl. The arrows are peak assignments where long arrows indicate stably trapped bases. (A) Full time trace of SERS spectra in the 45 nm diameter pore. (B) Comparison between SERS spectra of the λ-DNA in the 20 nm diameter pore and the 45 nm diameter pore. (C) Distribution of peak width of the SERS spectra from the 20 nm pore and the 45 nm pore. (D) Schematic of the folded λ-DNA of the 45 nm pore.

We remark that the quality of the Raman spectra from the 45 nm pores are not sufficiently high to draw conclusions. However, to better clarify the situation, we analyzed the average width of the Raman peaks for both pore sizes. The results are reported in Figure 3B,C where smaller pores are associated with narrow Raman peaks. It further confirms that for 20 nm pores the Raman signals mainly arise from single bases (narrower peaks), while for 45 nm pores Raman signals may come from a larger portion of the molecule (broader peaks). Notably, also for 45 nm pores we observed long trapping times of several seconds. However, no stable adenine peaks were observed in Raman spectra, which ruled out the possibility that the DNA segment was attached on the nanopore surface by the adenine interaction with gold.^12^

To better understand this trapping phenomenon, we investigated the electrofluidic behavior of the pore by means of numerical computations (COMSOL Multiphysics). We calculated the velocities of electro-osmotic (EO) ion flow and electrophoretic (EP) DNA flow and investigated the effect of having a pore surface with fixed vs floating charges. The results are shown in Figure 4 that compares the two configurations we simulated and the related EO and EP flows. The pore cavity of the bowl-shaped nanopore consists of 2 parts: (1) the Si_3_N_4_ cavity of 30 nm height and (2) the gold cavity of 5 nm height on top of the Si_3_N_4_ cavity. Si_3_N_4_ has negative surface charges that are fixed under electric bias. In opposition, gold has a positive surface charge that, being a conductor, can move upon bias application.^31^ Thus, the gold layer is polarizable under bias (see also Supporting Information Figure S6 for details). The application of an electric bias thus generated a bipolar electrode effect^20,21^ on the floating surface charge of the gold pore. As a result, a nontrivial EO sheath flow may be generated in the downward direction, as shown in Figure 4A, B. Namely, a faster flow is generated in the gold cavity center, while a slow flow occurred near the gold cavity sidewall. Most 4-ABT monolayers on the gold surface remain neutral in our experimental conditions, thus not affecting the EO sheath flow, as discussed in the Supporting Information (Note 4). Consequently, one should expect that negatively charged DNA molecules enter the Si_3_N_4_ cavity center (from backward) and shift toward the gold pore wall while exiting the pore (Figure 4B). An example of this trajectory is represented in Figure 4B with a white arrow. In contrast, the bipolar effect does not occur in a nanopore fully covered with fixed charges (Figure 4C), and the corresponding EO flow is uniform in the nanopore (Figure 4D).

**Figure 4:**
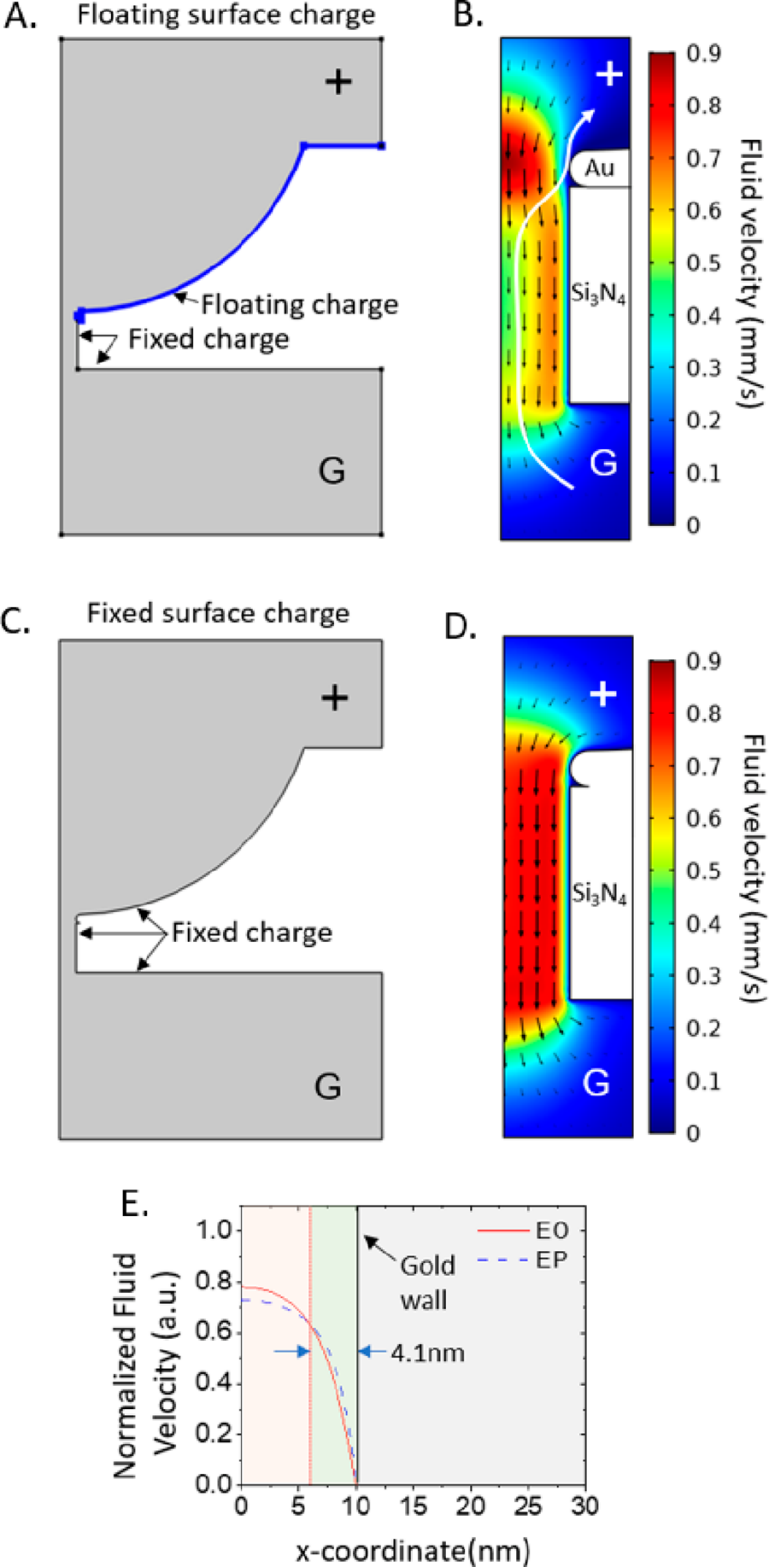
Multiphysics simulation of the electro-osmotic sheath flow and the trapping effect in the bowl-shaped nanopore. (A) Model of a 20 nm gold bowl-shaped nanopore with floating surface charge and its (B) simulated electro-osmotic sheath flow in the nanopore. The white arrow indicates the path favored by translocated DNA in the nanopore. (C) Model of a 20 nm bowl-shaped nanopore with fixed charge and its (D) simulated uniform electro-osmotic flow in the nanopore. (E) Simulated electro-osmotic (EO) and electrophoretic (EP) flow in the bowl-shaped nanopore with a diameter of 20 nm along the gold cavity radius in correspondence of the plasmonic hot spot. The green region (EP > EO) and pink region (EP < EO) indicate the regions favored and unfavored for the DNA flow through, respectively. “G” and “+” indicate the positions of the grounding and positive electrode.

To be quantitative, we report in Figure 4E the comparison between the EO and EP fluid velocities along the gold cavity radius in correspondence of the plasmonic hot spot in the 20 nm pore. We found that in close proximity of the gold pore wall there is a region of 4.1 nm in lateral size in which the EP velocity exceeds the EO one. This region corresponds to a channel that directs the DNA to the gold pore wall where the hot spot is located. The analysis clearly suggests a possible approach to manipulate the trajectory of the molecule inside the nanopore. The approach can be very effective in delivering the molecule toward the probing area of the plasmonic pore even when the pore is rather large. In fact, a similar calculation carried out for the 45 nm pore indicates a similar but less pronounced behavior. As shown in Supporting Information Figure S8, the analysis shows that the EO and EP fluid velocities are comparable inside the gold cavity. Thus, we expect that the net force in the *z* direction acting on the DNA molecule during the translocation (EP minus EO) should be quite weak.

These findings suggest a stick-and-go translocation of the DNA in the hot spot because local interactions in the *x* direction which are normally weak and unable to significantly affect the DNA trajectory can become dominant. For instance, a short-range ion-induced dipole interaction could occur between the DNA and the gold pore wall in the *x* direction. When the DNA is close to the gold pore wall in the hot spot, the negatively charged phosphate groups in its backbone would induce dipoles on the polarizable gold pore wall.^32^ The induced dipole could thus act as an attractive force between the DNA and the gold pore wall.^32,33^ Another possibility suggested in the literature is the optical force. However, according to our simulations, the optical force on the DNA chain was neglected. More detailed discussions about optical force are in the Supporting Information (Note 3).

In conclusion, we investigated theoretically and experimentally 3D bowl-shaped nanopores for SERS detection of λ-DNA molecules under electric bias. Our simulation shows that the 3D bowl-shaped nanopore can concentrate the incident laser energy in a single hot spot whose size is comparable to the molecular size along both the direction of translocation and the orthogonal direction. We experimentally delivered DNA molecules into the pore and observed strong SERS signals without the use of labels or other plasmonic transducers such as nanoparticles. According to the collected data, the plasmonic hot spot has a size of less than 3 nm along the direction of translocation. This enabled us to reveal DNA submolecular segments composed of only 7 bases. Such a spatial resolution is comparable to that of systems for DNA sequencing by means of electrical readout.

We care to note that no mechanism exists to rule out detection of DNA segments different from those in Supporting Information Table S5. Given sufficiently long measurements, we believe that other segments can be observed with similar probabilities. Electric recording was not demonstrated because resistive pulse measurements on the bowl-shaped nanopores proved to be noisy due to the high dielectric noise^34^ and photoinduced ionic noise.^35^ Although the noise can be overcome by PDMS passivation^34^ or using the Pyrex/Si_3_N_4_ substrate,^36^ the SERS time traces provide rich information on sequence, molecule conformation, and trapping time, which is particularly useful for single-molecule measurements in nanopores of diameter >10 nm.

In addition, we found stable trapping of DNA molecules for tens of seconds. By means of electrofluidic simulation, we provided a preliminary explanation of the trapping phenomenon. Although this finding needs further investigations, it suggests an effective approach to manipulate the analyte trajectory into plasmonic pores. In fact, thanks to this mechanism, a molecule flowing through the pore can be delivered toward the plasmonic hot spot, i.e., the probing area of the sensor.

In summary, we introduced and validated a novel type of plasmonic nanopore capable of nanofluidic manipulation and detection of single biomolecules which has high potentials for single-molecule analysis and in prospective even sequencing of polymeric molecules.

## Supporting Information Available

Materials, fabrication, Raman measurements and hydrogel uncoiling, simulation details and supplementary simulation results, consideration of optical force, impact of 4-ABT charge and pore diameter on electro-osmotic sheath flow, SERS band assignment, and λ-DNA sequence examples (PDF)
