# Supplementary material for: Plasmonic Bowl-Shaped Nanopore for Raman Detection of Single DNA Molecules in Flow-Through
Source: Nano Lett. 2023 Jun 1;23(11):4830–6. doi: 10.1021/acs.nanolett.3c00340 (PMC10273459; doi:10.1021/acs.nanolett.3c00340)
Supplement: Supplementary file 1 — nl3c00340_si_001.pdf [file nl3c00340_si_001.pdf]

Supporting Information

Plasmonic bowl-shaped nanopore for Raman detection of single DNA molecules in flow-through

Yingqi Zhao[1,2,+], Aliaksandr Hubarevich[1,+], Angela Federica De Fazio[1], Marzia Iarossi[1], Jian-An Huang[2]*, and Francesco De Angelis[1]*

[1]Istituto Italiano di Tecnologia, Via Morego 30, 16163, Genova, Italy. E-mail: francesco.deangelis@iit.it

[2]Faculty of Medicine, Faculty of Biochemistry and Molecular Medicine, University of Oulu, Aapistie 5 A, 90220 Oulu, Finland. E-mail: jianan.huang@oulu.fi

[+]Equal contribution.

Table of Content

Supplementary Note 1. Materials, fabrication, Raman measurement and hydrogel uncoiling

Supplementary Note 2. Simulation details and supplementary simulation results

Supplementary Note 3. Consideration of optical force

Supplementary Note 4. Impact of 4-ABT charge and pore diameter on electro-osmotic sheath flow

Supplementary Note 5. SERS band assignment and λ-DNA sequence examples

Supplementary Note 1. Materials, Fabrication, Raman measurement and hydrogel uncoiling

Materials

4-Aminobenzenethiol (4-ABT, Sigma-Aldrich 422967), Lithium chloride(Sigma-Aldrich, L9650) were brought from Sigma-Aldrich Co., LLC. Polydimethylsiloxane (PDMS, Dow Corning SYLGARD 184 silicone elastomer) was purchased from Dow Chemical Company. Si_3N_4 membranes (Norcada, NT005X TEM grid) were purchased from Norcada Inc.

Bowl-shaped nanopore Fabrication and encapsulation.

The gold bowl-shaped nanopores were fabricated on commercial Si_3N_4 membranes supported on silicon (Norcada, NT005X TEM grid). The size and thickness of the Si_3N_4 window was 0.05 × 0.05mm and 30nm respectively. After sputtering a 2 nm titanium and 100 nm gold layer on the front side of the Si_3N_4 membrane, FIB milling (FEI Helios NanoLab 650 DualBeam) at a voltage of 30 keV and a current of 7.7pA was used to sculpt bowl-shaped nanopore array at the front side of the gold film. The Array pitch is 5 µm. The bowl shape sculpturing pattern consists with a group of concentric ring patterns. The sculpture depth of each ring decreased from the center to the bowl edge and was adjusted to fine-tune the bowl shape profile and central pore size. Then 5-10nm of gold was sputter coated on the front side of the nanopore to adjust the central pore size and pore thickness. Scanning electron microscope was used to characterize the bowl-shaped nanopore morphology. Transmission electron microscopy (TEM) was used to confirm the final center pore diameter. To decorate the 4-ABT monolayer on the nanopore surface, the bowl-shaped nanopore samples were soaked in 10^{-4}M 4-ABT ethanol solution for 8h. Then the chip was rinsed with ethanol and DI water to remove the extra molecules. Then the bowl-shaped nanopore samples were embedded in a microfluidic chamber made from PDMS 80°C for approximately 20 min.

Hydrogel Preparation

Agarose was dissolved in 1× Tris–borate–EDTA buffer (89 mM Tris–borate; 2 mM EDTA; pH 8.3), with 1% as concentration at 80 °C. Before hydrogel layer preparation, the whole microfluidic device was treated with oxygen plasma to the hydrophilic surface. To form an agarose layer at the entrance of the bowl-shaped nanopore, 2ul melted gel solution was dropped at the lower reservoir of the Si_3N_4 membrane which is supporting the bowl-shaped nanopore and allow cool down naturally for 5min. Then the device was preserved in DI water to maintain hydrophilic.

Raman measurements

Raman measurements were obtained from a Renishaw inVia Raman spectrometer with a Nikon 60 × water immersion objective with a 1.0 NA illuminating a 785-nm laser beam and an exposure time of 0.5 s. The laser beam was focused to a spot diameter of 1.5 µm with a power varying from 2 to 12 mW. Raman measurements of λ-DNA flow through were done by collecting Raman signals at a nanopore for at least 5 minutes. 5 µg/ml λ-DNA dispersed in Tris–EDTA buffer was dropped in the lower reservoir of the membrane. 1M LiCl solution was used as the electrolyte, 30mV of voltage was

applied over the upper and lower part of the Si_3N_4 membrane with Pt wire electrode to drive the DNA from the bottom reservoir through the nanopore to the top reservoir.

Uncoiling the λ-DNA in the hydrogel

According to the "biased reptation model", the path of the DNA in the hydrogels can be described as a tube. When the λ-DNA gyration radius (500 – 600 nm) is larger than the hydrogel pore size, the DNA tends to unfold and linearize, as shown in Figure S1 below.[1, 2] This is due to the fact that, when electric bias is applied, all segments of the DNA in the hydrogel are subject to the electric forces. For the part of the tube (trajectory) not fully aligned in the direction of the electric field, the field itself will push the DNA segments against the hydrogel fibers and pull the folded segments of the DNA in Figure R1a out of the tube. The entropy barrier during this uncoiling process is overcame by the sufficient field strength in the hydrogel pores.

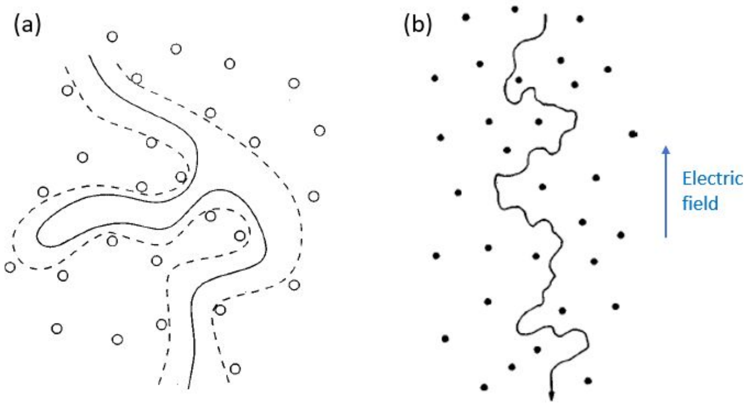

Figure S1. (a) Schematic representation of the DNA chain (solid line) and its ''tube'' (dotted lines) in the gel (bold circles). Note that this particular figure corresponds to a chain with a loop or folded segment in the gel pore. (b) the biased reptation model to uncoil a DNA chain in the gel (black dots) along the direction of the applied electric bias. Figures are from Ref.[1]

Supplementary Note 2. Simulation details and supplementary simulation results

We used the COMSOL Multiphysics® software to perform the optical and fluid dynamic simulations.[3]

For optical simulation, the refractive index and extinction coefficient of the gold was taken from Ref.[4] and the refractive index of the water and silicon nitride were set to 2.0 and 1.33, respectively. The incident light from 0.4 to 1 µm was irradiated along the Z axis to determine the plasmon resonance wavelength. The Gaussian source with a beam radius of 500 nm was used to illuminate a single bowl-shaped nanopore. The perfectly matched layers were applied along the X, Y and Z axes.

The bowl-shaped nanopore was constructed based on the Scanning electron microscope (SEM) images and cut to a unit cell with the simulation parameters listed in Figure S2 and Table S1.

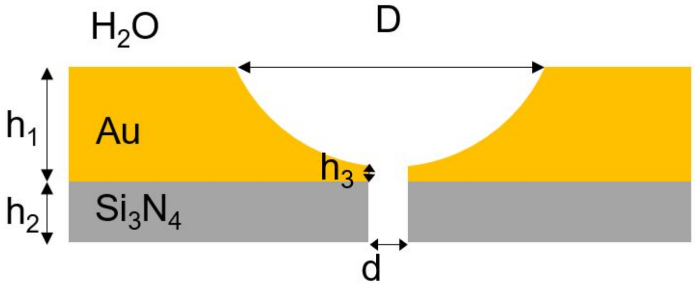

Figure S2. The dimension information of bowl-shaped nanopore structure measured from SEM images. h_1 *= 100nm,* h_2 *= 30nm,* h_3 *= 5-10 nm, D = 250 nm, d = 20-50nm.*

Table S1. Optical simulation parameters

Unit cell size	2×2×2.5 μm^3
Thickness of the bowl-shaped layer	100 nm
Thickness of silicon nitride	30 nm
Diameter of the hemisphere	250 nm
Diameter of the nanopore	from 2 nm till 50 nm
Thickness of the nanopore (metallic part)	from 2.5 nm till 10 nm

Comparison between conventional and bowl-shaped pores

As discussed in the main text, the bowl shape offers different advantages with respect the conventional shape (rectangular cross section). First, the bowl-shape has a single hot spot that provide a well-defined (unique) probing volume. In opposition, a rectangular pore has two hot spots: one lying on the upper surface and one in the bottom surface. These multiple hot spots, occurring also in porous materials, make the Raman analyses more complicated and potentially misleading. The comparison is shown in Figure S3. Second, the bowl-shape provides a much higher plasmonic intensity with no cut off with respect of the pore size. Indeed, the lower the radius the higher the enhancement. This is in opposition of conventional pores in which a reduction of radius or thickness implies a reduction of field enhancement. This comparison is reported in Figure S4.

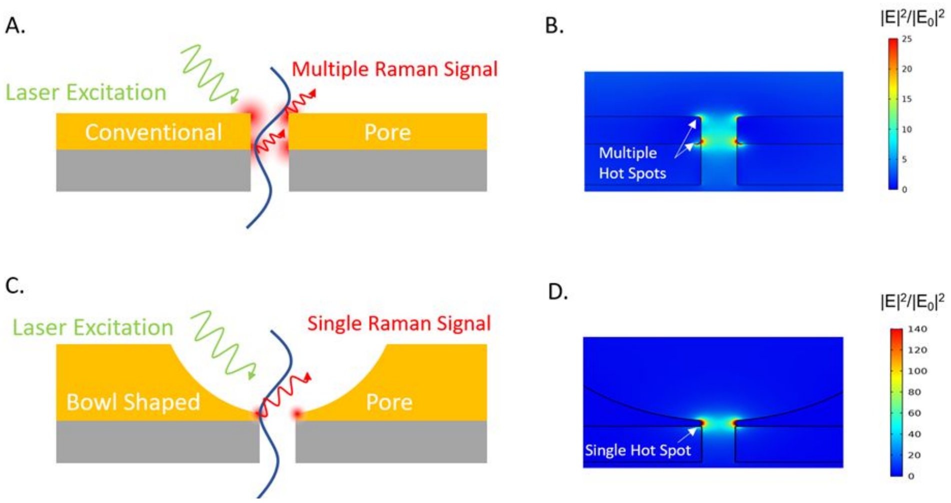

Figure S3. Scheme representing plasmonic nanopore and detection mechanism of (A) conventional pore and (C) bowl-shaped pore. Simulation of the electromagnetic field distribution in (B)conventional and (D)bowl-shaped pore. The colour bar indicates the electric field enhancement.

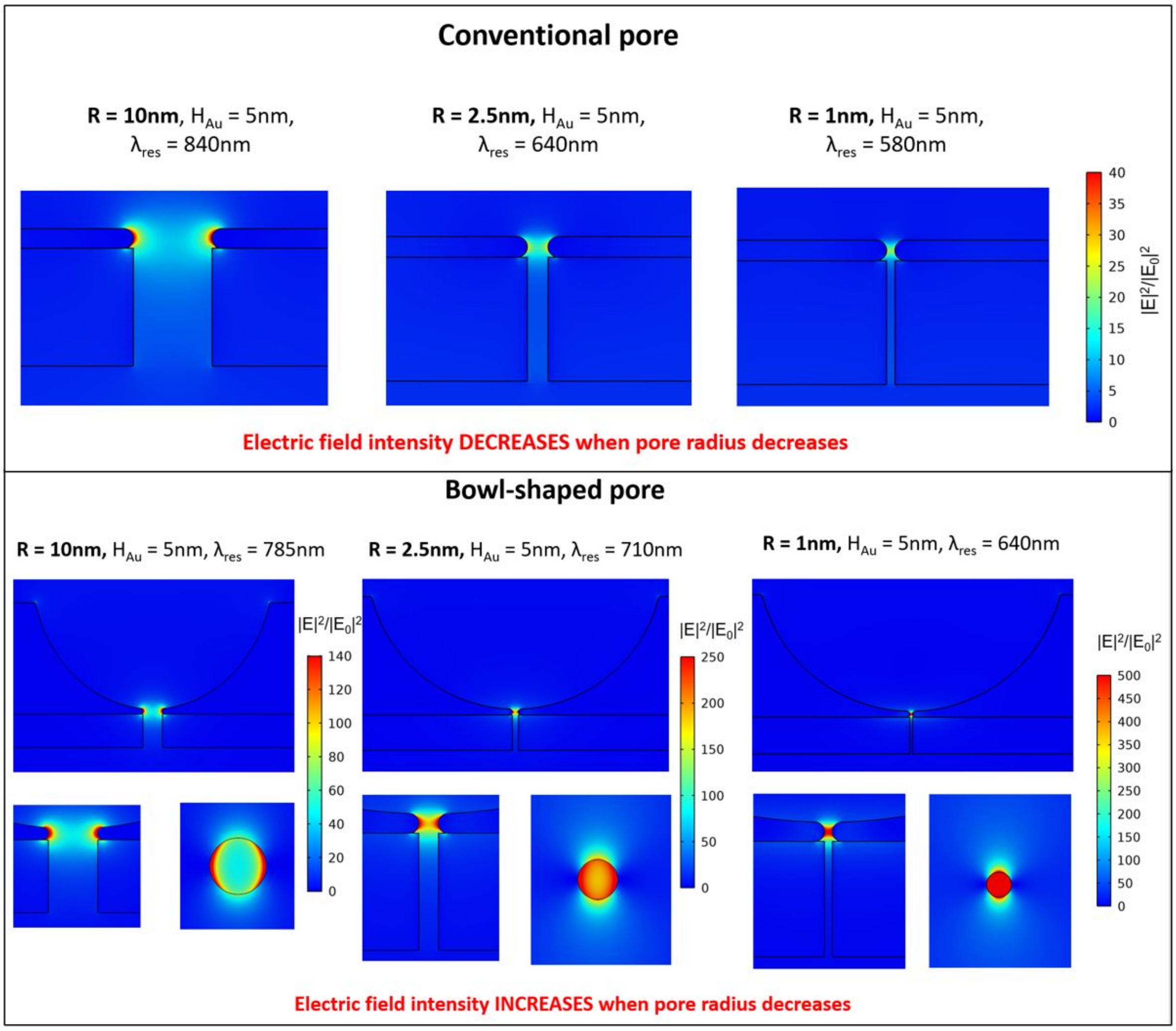

Figure S4. Simulated electric fields at resonance wavelength (λ_{res}) for conventional cylindrical gold nanopores (upper) and bowl-shaped gold nanopores (lower) with the same gold thickness (H_{Au}) at the nanopore edge but decreasing radius (R).

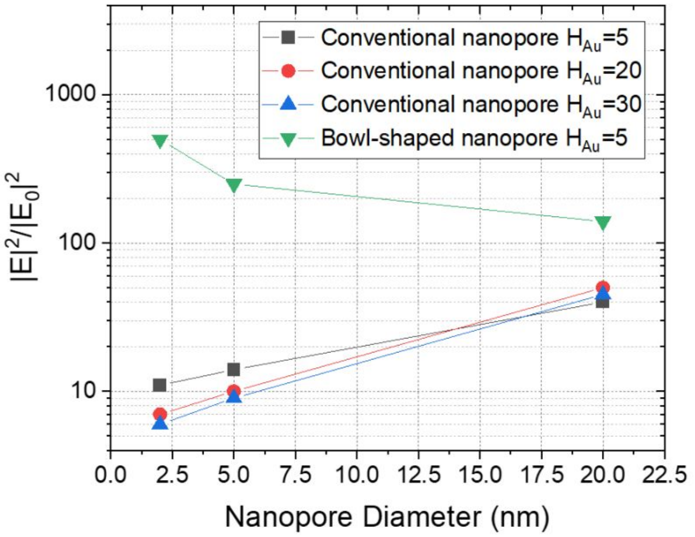

Figure S5. Simulated electric fields at resonance wavelength for conventional cylindrical gold nanopores and bowl-shaped gold nanopores with different diameters and gold thicknesses (H_{Au}).

For fluid dynamics simulation, we used three physics to perform the fluid dynamics simulation[5]:1. The electrostatics physics for solving for the electric potential. 2. The transport of diluted species interface for solving the species concentrations. 3. The laminar flow interface for solving for the velocity field and the pressure. The above-mentioned physics are connected to each other using multiphysics coupling features: i) space charge density coupling, ii) reacting flow, diluted species and iii) potential coupling. The constant charge was applied to the dielectric interface, while the floating potential of zero initial value of voltage was applied to the metallic interface.[6] The axial symmetric boundary conditions are applied on the axis of the bowl- shaped nanopore. The surface charge density of the silicon nitride in the fluid was set to -0.15 C/m^2.[7] The DNA was modeled as a cylinder rod with a 2nm diameter and surface charge density of -0.15 C/m^2.[8] The simulation parameters of the fluid are given in Table S2.

Table S2. Fluid simulation parameters

Relative permittivity	80
Density	1000 kg/m^3
Dynamic viscosity	1 mPa/s
Bulk concentration	1M
Diffusivity of K+	1.95e-9[m^2/s]
Diffusivity of Cl-	2.03e-9[m^2/s]
Valence of K+	1
Valence of Cl-	-1

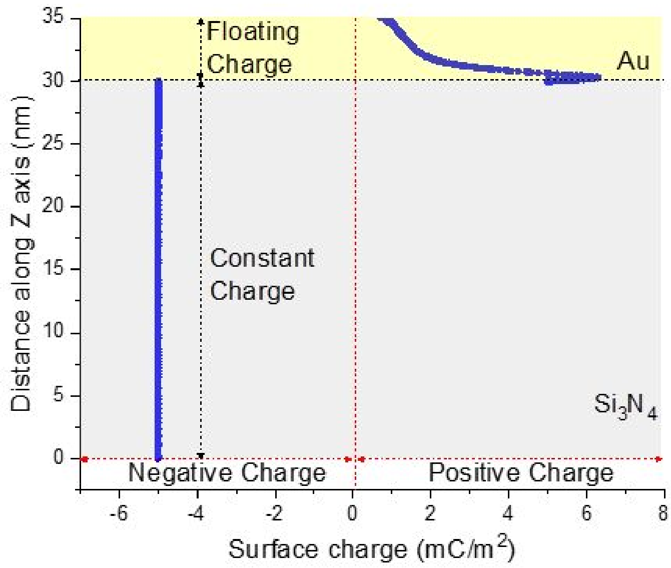

Figure S6. Multiphysics simulation of the surface charge in the bowl-shaped nanopore on silicon nitride.

Supplementary Note 3. Consideration of Optical Force

To calculate the optical force, DNA was treated as a particle of 2nm diameter or cylinder 2nm × 5nm. The gap between particle and pore side wall was assumed to be 1nm. The optical force was calculated by Maxwell stress tensor and summarized in the table below:

Table S3. Optical force calculated by Maxwell stress tensor

Particle Size and Shape	F_x	F_z
Cylinder of 5nm×2nm	0.2fN	0.3fN
Sphere of 2nm	0.06fN	0.09fN

According to the literature, one necessary condition for optical trapping is that the potential energy of the gradient optical force is much larger than the Brownian motion kinetic energy:[9]

$$\exp\left(\frac{U}{k_b T}\right) \gg 1 \quad \text{(S1)}$$

Where U is the potential energy of the gradient optical force $U = \frac{4n_2R^2}{c}\left(\frac{\varepsilon_1 - \varepsilon_2}{\varepsilon_1 + 2\varepsilon_2}\right)\frac{P}{w_0^2}$, in which k_b is the Boltzmann constant, T is the temperature of the environment around nanoparticles, n_2 is the refractive index of the environment, R is the radius of the nanoparticles, c is the speed of light, ε_1 and ε_2 are the dielectric constants of the nanoparticle and environment respectively, P is the laser power, and w_0 is the waist radius of laser beam. In our case, DNA was treated as approximately a nanoparticle with 2nm diameter, the estimated $U/(k_bT)$ is about 10^{-4}, which means that the optical force cannot be stronger than the Brownian one. Therefore, the electro-osmontic and electrophoretic forces are the key forces for moving the λ-DNA to the hotspot.

Supplementary Note 4. Impact of 4-ABT charge and pore diameter on electro-osmotic sheath flow

According to Crooks' work,[10] 4-ABT attached to Au surface will have a positive charge at low pH and a neutral charge at high pH. The charging status will also be influenced by electrode potential. pKa of 4-ABT on the gold surface is 6.9, at +0.2V. So at pH near 8(the buffer we used), most of the 4-ABT are neutral, with a small amount still keeping a positive charge. The precise neutralization ratio is also influenced by the electrode potential. As shown in Figure S7, a positive charge at the top gold layer surface and a negative charge at the bottom silicon nitride layer results in the formation of sheath flow. Therefore the 4-ABT layer on the gold will not prevent the formation of sheath flow.

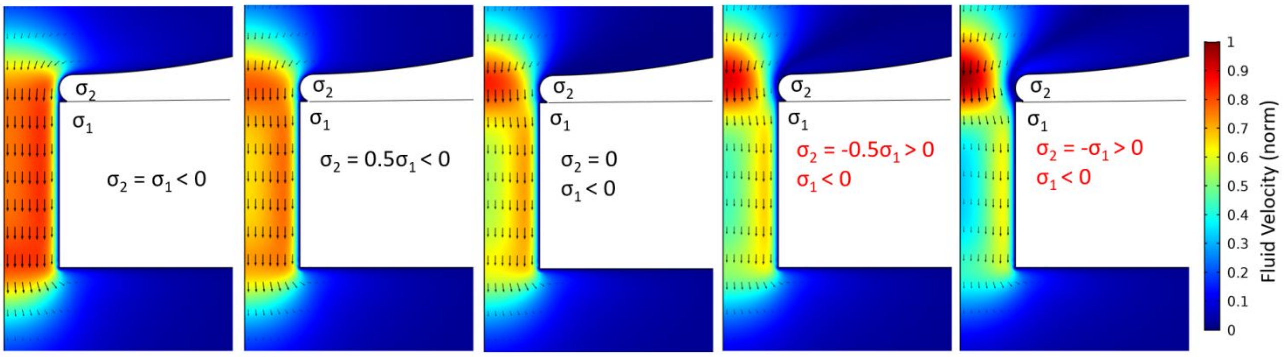

Figure S7. Multiphysics simulation of the influence of surface charge in the 20 nm bowl-shaped nanopore on silicon nitride and gold layer. The color bar indicates the fluid velocity. A positive charge at the top gold layer surface and a negative charge at the bottom silicon nitride layer results in the formation of sheath flow which brings DNA close to the hot spot at the edge of the gold layer.

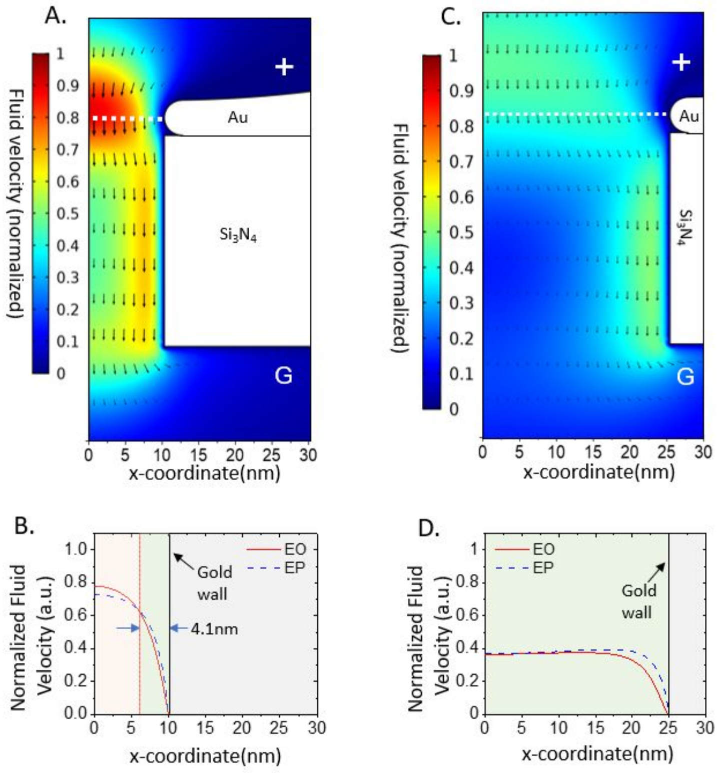

Figure S8. Comparison of the simulated electro-osmotic sheath flow and the nanofluidic trapping effect in the bowl-shaped nanopore of 20 nm and 50 nm in diameter. (A, C) simulated electro-osmotic sheath flow in the bowl-shaped nanopore with a diameter of (A)20 nm and (C) 50 nm. The white dotted lines indicate the positions where the corresponding normalized fluid velocity distributions of the electrophoretic (EP) DNA flow and electro-osmotic (EO) flow were calculated in (B, D). The green region (EP>EO) and pink region (EP<EO) indicate the region favored and unfavored for the DNA flow through respectively. The color bars indicate the EO fluid velocity. "G" and "+" indicate the positions of the grounding and positive electrode.

Supplementary Note 5. SERS band assignment and λ-DNA sequence examples

Table S4. Assignment of the SERS bands of the 4-ABT and DNA in Figure 2 and 3

Molecule	Observed SERS modes (cm^{-1})	Most probable assignment[a] with reference
4-ABT on Nanopore	1078	str C-S[11]
	1583	str C-C[11]
Adenine (A)	1321	str C5−N7, N1−C2, C2−N3, C5−C6, bend C2/8-H[12]
Thymine (T)	1184	bend C6-H, strC2-N3[13]
	1220-1224	str C5−C9[12]
	1528	ring str[12]
	1608	str C2=O, C4=O[12]
Cytosine (C)	802	ring breath[12]
	1307-1314	ring str C−N[12]
	1430	bend N1−H, C5−H, C6−H[12]
Guanine (G)	527	6-ring def[12]
	584	6-ring def[12]
	1141-1153	rock NH_2, ring str C−N[12]
	1233-1234	bend C8−H, str N5−N7, N7−C8[12]
	1300	ring str C−N, C−C, bend C8−H, rock NH_2 [12]
	1353	bend N1−H, N10−H12, str C2−N10 [12]
	1389	ring str C−N, C−C, rock NH_2, bend N1/9−H- [12]
DNA Backbone (B)	830	str O – P – O [14]
	889	deoxyribose ring [14]

[a] Abbreviations: bend: bending; breath: breathing; def: deformation; rock: rocking; str: stretching; 6-ring: six-membered ring

Table S5. Summary of sequence examples in bold in lambda DNA from a database: https://www.addgene.org/browse/sequence_vdb/6234/ according to the SERS spectra in Figure 2.

Figure	Possible Sequence examples	Matching sequences in Lambda DNA	Sequence positions
2B	Stable C, fluctuating T: TCCCCCT	GGGC**TCCCCC T**GTCGGTGCA	585-591
		GCATCA**TCCC CCT**GTTCGAC	31967-31973
		AC**AGGGGGA**C ACAAAAGACA	39113-39119: reverse complement
		ACGCCGG**AGG GGGA**AAGATA	14218-14224: reverse complement
		GCTGAA**AGGG GGA**TGTATGG	7967-7973: reverse complement
2D 1)	Stable T, fluctuating	TTTTT**GTTTT TTG**AATAAT	23767-23773

	G: GTTTTTTTG	AA**CAAAAAAA C**CAACAGCATA	38223-38231: reverse complement
2D 2)	Stable G, fluctuating A: AGGGGGA	GGGC**TCCCCC T**TGTCGGTGCA	585-591: reverse complement
		GCTGAA**AGGG GGA**TGTATGG	7967-7973
		ACGCCGG**AGG GGGA**AAGATA	14218-14224
		AC**AGGGGGA**C ACAAAAGACA	39113-39119
		GCATCA**TCCC CCT**GTTCGAC	31967-31973: reverse complement

References

(1) Viovy, J. L. Electrophoresis of DNA and other polyelectrolytes: Physical mechanisms. *Reviews of Modern Physics* **2000**, *72* (3), 813-872. DOI: 10.1103/RevModPhys.72.813.
(2) Tang, Z. P.; Liang, Z. X.; Lu, B.; Li, J.; Hu, R.; Zhao, Q.; Yu, D. P. Gel mesh as "brake" to slow down DNA translocation through solid-state nanopores. *Nanoscale* **2015**, *7* (31), 13207-13214. DOI: 10.1039/c5nr03084f.
(3) *COMSOL Multiphysics® v. 6.0 www.comsol.com. COMSOL AB, Stockholm, Sweden*. Date of access: 23 May 2023.
(4) Rakic, A. D.; Djurisic, A. B.; Elazar, J. M.; Majewski, M. L. Optical properties of metallic films for vertical-cavity optoelectronic devices. *Applied Optics* **1998**, *37* (22), 5271-5283. DOI: Doi 10.1364/Ao.37.005271.
(5) COMSOL Blog. Modeling Electroosmotic Flow and the Electrical Double Layer, 28 October **2013**. https://www.comsol.com/blogs/modeling-electroosmotic-flow-electrical-double-layer/. Date of access: 23 May 2023.
(6) Zhang, M.; Ai, Y.; Sharma, A.; Joo, S. W.; Kim, D.-S.; Qian, S. Electrokinetic particle translocation through a nanopore containing a floating electrode. *ELECTROPHORESIS* **2011**, *32* (14), 1864-1874. DOI: https://doi.org/10.1002/elps.201100050.
(7) Lin, K.; Li, Z.; Tao, Y.; Li, K.; Yang, H.; Ma, J.; Li, T.; Sha, J.; Chen, Y. Surface Charge Density Inside a Silicon Nitride Nanopore. *Langmuir* **2021**, *37* (35), 10521-10528, Article. DOI: 10.1021/acs.langmuir.1c01504.
(8) Jia, Z.; Choi, J.; Park, S. Surface Charge Density-Dependent DNA Capture through Polymer Planar Nanopores. *Acs Applied Materials & Interfaces* **2018**, *10* (47), 40927-40937. DOI: 10.1021/acsami.8b14423.
(9) Harada, Y.; Asakura, T. Radiation forces on a dielectric sphere in the Rayleigh scattering regime. *Optics Communications* **1996**, *124* (5-6), 529-541, Article. DOI: 10.1016/0030-4018(95)00753-9.
(10) BRYANT, M.; CROOKS, R. DETERMINATION OF SURFACE PKA VALUES OF SURFACE-CONFINED MOLECULES DERIVATIZED WITH PH-SENSITIVE PENDANT GROUPS. *Langmuir* **1993**, *9* (2), 385-387, Letter. DOI: 10.1021/la00026a005.
(11) Kim, K.; Yoon, J. Raman scattering of 4-aminobenzenethiol sandwiched between Ag/Au nanoparticle and macroscopically smooth Au substrate. *Journal of Physical Chemistry B* **2005**, *109* (44), 20731-20736, Article. DOI: 10.1021/jp052829b.
(12) Madzharova, F.; Heiner, Z.; Guhlke, M.; Kneipp, J. Surface-Enhanced Hyper-Raman Spectra of Adenine, Guanine, Cytosine, Thymine, and Uracil. *Journal of Physical Chemistry C* **2016**, *120* (28), 15415-15423, Article. DOI: 10.1021/acs.jpcc.6b02753.
(13) OTTO, C.; VANDENTWEEL, T.; DEMUL, F.; GREVE, J. SURFACE-ENHANCED RAMAN-SPECTROSCOPY OF DNA BASES. *Journal of Raman Spectroscopy* **1986**, *17* (3), 289-298, Article. DOI: 10.1002/jrs.1250170311.
(14) Benevides, J.; Overman, S.; Thomas, G. Raman, polarized Raman and ultraviolet resonance Raman spectroscopy of nucleic acids and their complexes. *Journal of Raman Spectroscopy* **2005**, *36* (4), 279-299, Review. DOI: 10.1002/jrs.1324.
